# A cross-sectional study of vascular risk factors in a rural South African population: data from the Southern African Stroke Prevention Initiative (SASPI)

**DOI:** 10.1186/1471-2458-7-326

**Published:** 2007-11-13

**Authors:** Margaret Thorogood, Myles Connor, Stephen Tollman, Gillian Lewando Hundt, Gerry Fowkes, Jennifer Marsh

**Affiliations:** 1 University of Warwick, Coventry, UK; 2 University of the Witwatersrand, Parktown, Johannesburg, South Africa; 3 University of Edinburgh, Teviot Place, Edinburgh, UK

## Abstract

**Background:**

Rural sub-Saharan Africa is at an early stage of economic and health transition. It is predicted that the 21^st ^century will see a serious added economic burden from non-communicable disease including vascular disease in low-income countries as they progress through the transition. The stage of vascular disease in a population is thought to result from the prevalence of vascular risk factors. Already hypertension and stroke are common in adults in sub-Saharan Africa. Using a multidisciplinary approach we aimed to assess the prevalence of several vascular risk factors in Agincourt, a rural demographic surveillance site in South Africa.

**Methods:**

We performed a cross sectional random sample survey of adults aged over 35 in Agincourt (population ≈ 70 000). Participants were visited at home by a trained nurse who administered a questionnaire, carried out clinical measurements and took a blood sample. From this we assessed participants' history of vascular risk, blood pressure using an OMRON 705 CP monitor, waist circumference, body mass index (BMI), ankle brachial index (ABI), and total and HDL cholesterol.

**Results:**

402 people (24% men) participated. There was a high prevalence of smoking in men, but the number of cigarettes smoked was small. There was a striking difference in mean BMI between men and women (22.8 kg/m^2 ^versus 27.2 kg/m^2^), but levels of blood pressure were very similar. 43% of participants had a blood pressure greater than 140/90 or were on anti-hypertensive treatment and 37% of participants identified with measured high blood pressure were on pharmacological treatment. 12% of participants had an ABI of < 0.9, sugesting the presence of sub-clinical atheroma. 25.6% of participants had a total cholesterol level > 5 mmol/l.

**Conclusion:**

We found a high prevalence of hypertension, obesity in women, and a suggestion of subclinical atheroma despite relatively favourable cholesterol levels in a rural South African population. South Africa is facing the challenge of an emerging epidemic of vascular disease. Research to establish the social determinates of these risk factors and interventions to reduce both individual and population risk are required.

## Background

Vascular disease is a major cause of morbidity and mortality throughout the world, although its manifestations differ in countries at different stages of economic development. This has been encapsulated in the theory of health transition [1-3] that describes a continuum where an early stage is characterised by hypertensive heart disease and a large proportion of haemorrhagic stroke. Later on, driven by increasing prevalence of vascular risk factors in the population[3], ischaemic heart disease and ischaemic stroke due to atherosclerosis emerge[1,3]. Rural sub-Saharan Africa is at an early stage of economic and health transition [4] but there is currently very little published information on the prevalence of vascular risk factors in the population, although such data will be essential in planning future health services. Several recent reports have predicted that the 21^st ^century will see a serious economic burden from non-communicable disease in low income countries [4,5], and have called for action. Hypertension as a vascular risk factor and stroke as a vascular outcome are important in adults in sub-Saharan Africa [1,3,6-13]

The high mortality from stroke in Agincourt sub-district between 1992 and 1995 [10] led to the Southern Africa Stroke Prevention Initiative (SASPI) [11,12]. This multidisciplinary project aimed to describe and address the burden of stroke and other vascular disease in rural Sub-Saharan Africa. Among other projects, it included a cross-sectional survey of the prevalence of vascular risk factors in the adult population of Agincourt, which we report here.

The aim of this paper is to describe the prevalence of risk factors for vascular disease in a rural sub-Saharan population aged over 35 years.

## Methods

### Setting and Population

The Agincourt sub-district is in South Africa's rural north-east, adjacent to Mozambique, where the Wits/MRC (Agincourt) Rural Public Health and Health Transitions Research Unit has been monitoring causes of death, births and migration in a population of around 70 000 people since 1992[14]. Each individual has a unique identity number, as does each household. Data is updated annually by census, with trained field workers visiting every household.. This dataset therefore provides a well categorised sampling frame for studies such as this.

The sub-district has 21 villages of varying sizes and is heavily populated at about 170 persons per sq km. Electricity is available in most villages, but not all households. At the time the study was undertaken, some villages had no standpipe to provide clean water and supply from standpipes was limited and sporadic in all other villages. There is high unemployment and, as is common in rural sub-Saharan Africa, considerable labour migration, especially in men. Household plots are too small for subsistence agriculture, although crops supplement the diet [15].

Most people access plural health care from allopathic health professionals, traditional healers, and prophets linked to churches that offer faith based healing[16]. There are five publicly funded primary care clinics and a large health centre staffed by nurses, with occasional visits from doctors. Treatment and drugs are free at these clinics. Three district hospitals serve the wider district, and there are a number of private practitioners.

### Sample

We stratified the Agincourt villages by size (less than 500 households, and 500 households or more) and by whether the village was a formal settlement (officially recognised) or an informal settlement (settled following an influx of refugees during the Mozambican civil war). Ten villages were randomly selected: four small informal villages, three small formal villages, and three large formal villages (there were no large informal villages). The total population of the selected villages was 28,715, representing 43% of the population of the sub-district. A random sample of individuals from the resident (i.e. excluding migrant workers) population in selected villages aged 35 years and older, provided 526 individuals. This was approximately 10% of the population aged 35 years and over in the selected villages, or 3% of the 16, 705 sub-district population aged 35 years and over). We considered migrant workers or temporary migrants to be people who were linked to a household and provided financial support but lived in the area for less than 6 months of the year because they were employed elsewhere.

Information on age, gender and the household asset score was available from the Agincourt database. The household asset score was based on information collected during the 2001 census about the type and size of dwelling; access to water and electricity; appliances and livestock owned and transport available. The questionnaire contained 34 variables, which were developed in discussion with local field staff and community members and the score was developed using a principal component factor analysis. The score was divided into quintiles labeled: low, medium-low, medium, medium-high and high economic status[17]. A similar household economic status index was reported to discriminate effectively among levels of economic status in other rural African settings[18].

### Making contact with participants

We made at least three attempts to visit each individual. A second sample provided substitutes for individuals who were ineligible (for example had died, were migrant workers, were aged < 35 years, or were not living there) or who were untraceable. Subjects who declined to participate, were too ill, or did not keep appointments were not replaced. In three households in a remote village the interview was abandoned for logistic reasons.

### Consent and ethics

We obtained informed consent from the village headman and through village community meetings, before starting work in each village. In addition, each participant was asked to consent separately in writing to each component of the study, (the interview, the anthropometric and blood pressure measurements, and venesection). Many participants were illiterate and marked the form with a thumbprint, but we ensured they understood the content of the form. Ethics committee approval was granted by both the London School of Hygiene and Tropical Medicine (755) and the University of the Witwatersrand (M02-04-63).

### Interview and clinical examination

In 12 months from July 2002 two specially trained senior nurses from the local community carried out interviews and clinical examinations. The interview covered a past medical history, including hypertension, and smoking habit. Participants were asked about any treatment for high blood pressure in the last week, and asked to show the medication to confirm the name.

The nurses were trained in anthropometric and blood pressure measurement by a member of staff from the Medical Research Council, which carried out the South African Demographic and Health Survey [19]. They measured height using a portable, collapsible stadiometer (Leicester Height Measure – Seca, Ltd, Birmingham, UK), which was placed on a firm level surface. They then asked participants to remove any headdress and footwear, to stand on the stadiometer platform with their heels together touching the backstop to ensure that their spine at pelvis and shoulder level, as well as the back of their head touched the upright rod of the stadiometer. The nurse then positioned the participant's head so that an imaginary horizontal line joined the corner of the participant's eye and the upper attachment of their ear to their head. They then firmly lowered the measuring arm of the stadiometer onto the participant's head without forcing their head down. Participants were then asked to move away from the stadiometer and the nurse read the measurement to the nearest 0.1 cm.

For waist measurement, the nurse asked the participants to stand upright in a relaxed manner with their arms slightly away from their sides and their feet about 15 cm apart. The nurse then placed the measuring tape around the narrowest part of the bare waist, 2.5 cm above the umbilicus. In difficult cases, for instance, obese persons, the nurse stood behind the participant to find the narrowest part of the waist. If this was not the narrowest portion of the waist, they then measured the smallest circumference between the xiphi sternum and umbilicus. They took the measurement with the measuring tape in a horizontal position to the nearest 0.1 cm after normal expiration, taking care not to insert their fingers under the tape and not to let the tape cut into the participant.

Participants were weighed barefoot and wearing at most light clothing using a UC-321 Precision Health Scale UC-321 scales (A&D Medical). The scale was placed on a flat surface and cleaned with disinfectant. A clean paper towel was placed on top of the scale prior to each measurement. The nurse then switched the scale on and ensured that it read zero. They then asked the participant to step onto the scale and stand with their feet firmly on the middle of the scale without moving while the nurse recorded the reading to the nearest 0.1 kg.

Sitting blood pressure was measured in the left arm using an OMRON 705CP blood pressure monitor and an appropriately sized cuff after the participant had been sitting for five minutes with their arm supported. Three measures were taken two to three minutes apart. Supine arm blood pressure was measured with the same monitor after the participant had been lying for 2 to 3 minutes. Ankle systolic pressures were measured in the posterior tibial artery of both legs using a Doppler ultrasound probe (Huntleigh Mini-Dopplex) and a Mandaus manual digital blood pressure monitor (PMS instruments) with the cuff placed just above the malleoli.

When the nurse identified any problem requiring treatment she gave the participant a letter of referral for the local clinic.

We assessed the quality of the anthropometric measures on several occasions. About midway through the study the MRC nurse trainer visited the site and accompanied the nurses on their visits, assessing their techniques and re-training as required. Several of us (MT, ST, GH and MC) visited the site and accompanied the nurses resulting in approximately monthly quality assessments, and one of us (MC) was available at least fortnightly on site to manage any queries from the nurses.

### Blood Analysis

Blood samples were transported in cold boxes, centrifuged locally at the end of each day, and transferred by courier to the University of the Witwatersrand Contract Laboratory Services. Analysis was done using the Roche Cobas Integra 400 System. Total and HDL cholesterol were measured using an enzymatic, colorimetric method,

### Clinical Definitions

We used the mean of the second and third blood pressure measurements and the Joint National Committee definition of hypertension (JNC7) [20] criteria to define hypertension. A person was considered to have high blood pressure if the systolic pressure was at or greater than 140 mmHg, or the diastolic at or greater than 90 mmHg, or they were using anti-hypertensives. As with JNC7, we further divided high blood pressure into Stage 1 (systolic 140–159 mmHg, and/or diastolic 90–99 mmHg) and Stage 2 (systolic 160 mmHg or higher, and/or diastolic 100 mmHg or higher).

In developed populations, the ankle brachial index is a good marker of subclinical peripheral arterial disease and an indicator of overall atherosclerosis[21]. A cut-off of the ankle brachial index of 0.9 is commonly used to distinguish individuals at high and low risk of clinical atherosclerosis. We used the supine systolic pressure together with the pressure as measured at the ankle (see above) to calculate the ankle brachial index in each leg. We have reported the lowest of the two ankle brachial indices, together with the proportion of participants with an ankle brachial index at or less than 0.9.

#### Physical activity

At the time, we were unable to find a validated physical activity questionnaire that was suitable for use in this environment where we found no concept of leisure time physical activity, and where walking carrying a load formed a major part of daily activity. We therefore adapted the validated International Physical Activity Questionnaire (IPAQ)[22], and categorised respondents as moderately active, low active or sedentary on the basis of their responses. Table 1 provides details of how respondents' physical activity was categorised.

**Table 1:** Definitions of physical activity categories

**Moderate Activity**	
Any one of:	Has a job which involves at least moderate activity
	Walks with heavy loads and/or cycles for at least 2.5 hours a week
	Does 1 hour or more of vigorous non-work activity, e.g. football
	Has a job that involves standing and walking AND Either walks with loads and/or cycles for at least 1 hour, or walks without loads for at least 2.5 hours a week.
**Low Activity**	Not sedentary but not active enough to fit the vigorous or moderate activity categories
**Sedentary**	Had no job, or a job that only involves sitting and does no walking, cycling, or vigorous non-work activity.

### Data analysis

We used STATA version 8 [23]. Categorical data was tabulated and confidence intervals for cell percentages calculated using the cii function. Differences in frequencies were examined using chi-squared test of the hypothesis that rows and columns are independent. Continuous clinical measurements were examined and summarised via their means and 95% confidence intervals.

## Results

### Response

**Figure 1:**
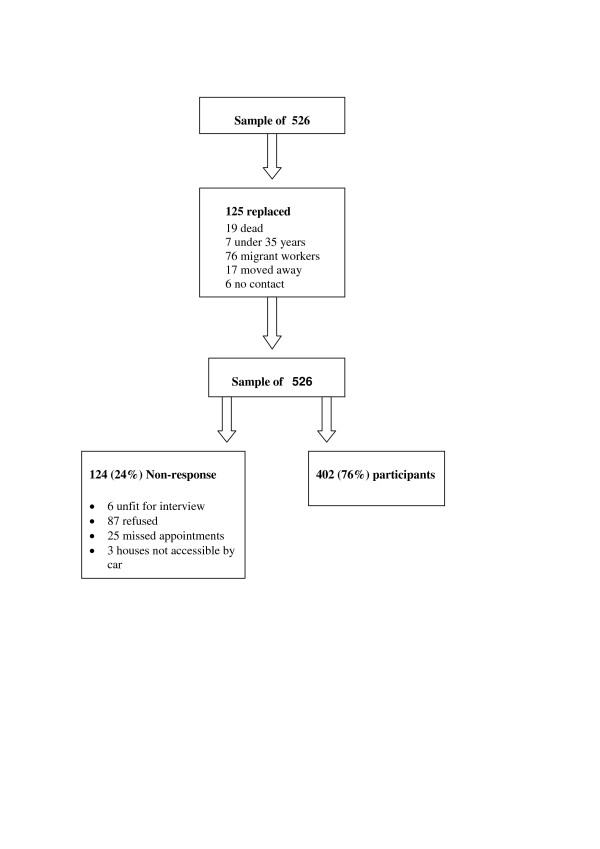
Replacements and final response rate.

Four hundred and two people (24% men) participated; a response rate of 77% (80% women, 68% men). At the mid year 2003 census update there were around 12 500 people aged over 34 resident in the field site of whom 32% were men, reflecting the dominance of male labour migration (Collinson M, personal communication). There was little difference in demographic variables in the total sample and in the responders (Table 2). Figure 1 shows the people who were replaced in the sample, and reasons for non-response in the valid sample. The response rate to different parts of the interview and examination varied: the lowest response rate was for venepuncture (51%).

**Table 2:** Comparison of demographic characteristics of total sample and participant sample.

**Variable**	**Total sample (N = 525)**	**Participant sample (N = 402)**
Female	73.3 (69.3,77.0)	76.4 (71.9,80.4))
Age category (years)		
35–44	29.5 (25.7,33.6)	28.9 (24.5, 33.6)
45–54	26.3 (22.6, 30.3)	27.9 (23.5, 32.5)
55–64	18.1 (14.9, 21.7)	17.9 (14.3, 22.0)
65–74	15.1 (12.1, 18.4)	13.4 (10.3, 17.2)
75 plus	11.0 (8.5, 14.0)	11.7 (8.7, 15.2)

Household asset score*		
1 (low)	12.5 (9.80, 15.7)	13.3 (10.2, 17.1)
2	177 (14.5, 21.3)	15.4 (12.0, 19.3)
3	29.9 (26.0, 34.0)	30.5 (26.0, 35.3)
4	20.4 (17.0, 24.2)	22.9 (18.9, 27.4)
5 (high)	19.5 (16.1, 23.1)	17.9 (14.2, 22.0)

Numbers shown are percentages and 95% confidence intervals.

* A score based on type and size of dwelling; access to water and electricity; appliances and livestock owned and transport available.

### Characteristics of the responder population

Table 3 shows the distribution of some lifestyle factors by gender. There was a high prevalence of smoking in men (28.4%), but the amount smoked was low. For many men smoking was too irregular to estimate a daily amount, and most of the other men reported 5 or less cigarettes daily. Money was limited and few people could afford the cost of regular smoking. There were also differences between men and women in drinking alcohol (Table 3).

**Table 3:** Distribution of demographic and lifestyle factors by gender

**Variables**	**Male N = 95**	**Female N = 307**	**Total N = 402**	**Significance of difference (male v female)**
	23.6 (19.6, 28.1)	76.4 (71.9,80.4)	100	
Age group				
35–44 years	16.8 (9.94, 25.9)	32.6 (27.4, 38.1)	28.9 (24.5, 33.6)	
45–54 years	29.5 (20.6, 39.7)	27.4 (22.5, 32.7)	27.9 (23.5, 32.5)	
55 -64 years	23.2 (15.1, 32.9)	16.3 (12.3, 20.9)	17.9 (14.3, 22.0)	d.f.= 4
65–74 years	11.6 (5.92, 19.8)	14.0 (10.3, 18.4)	13.4 (10.3, 17.2)	p = 0.007
75 plus years	18.9 (11.6, 28.3)	9.45 (6.42, 13.3)	11.7 (8.72, 15.2)	
Household asset score				
Low	14.7 (8.29, 23.5)	12.9 (9.34, 17.2)	13.4 (10.2, 17.1)	
Medium -low	21.1 (13.6, 30.6)	13.6 (9.92, 18.0)	15.4 (12.0, 19.3)	
Medium	25.3 (16.9, 35.2)	32.1 (26.9, 37.7)	30.5 (26.0, 35.3)	d.f. = 4
Medium-high	22.1 (14.2, 31.7)	23.2 (18.5, 28.4)	22.9 (18.9, 27.4)	p = 0.401
High	16.8 (9.94, 25.9)	18.2 (14.0, 23.0)	17.9 (14.2, 22.0)	
Currently smoking at least daily	28.4 (19.6, 38.6)	10.7 (7.50, 14.8)	14.9 (11.6, 18.8)	d.f. = 1 p < 0.001
Has never drunk alcohol	28.3 (19.4, 38.6)	74.9 (69.6, 79.7)	63.9 (59.0, 68.7)	
Has drunk alcohol but not as much as once a week in the last 12 months	14.1 (7.74, 23.0)	11.7 (8.29, 15.9)	12.3 (9.19, 15.9)	d.f. = 2
Drinks alcohol at least once a week in the last 12 months	57.6 (46.9, 67.9)	13.4 (9.73, 17.8)	23.8 (19.6, 28.3)	p < 0.001
Physical activity				
Moderately active	68.4 (58.1, 77.6)	74.9 (69.7, 79.7)	73.4 (68.8, 77.6)	
Low active	20.0 (12.5, 29.5)	18.2 (14.1, 23.0)	18.7 (15.0, 22.8)	d.f. = 2
Sedentary	11.6 (5.92, 19.8)	6.84 (4.28, 10.3)	8.0 (5.5, 11.1)	p = 0.275

Numbers shown are percentages and 95% confidence intervals.

*Only a small minority of smokers were able to estimate a daily number smoked

Tables 4 shows the distribution of some clinical measurements associated with vascular risk by gender, and Table 5 shows the proportion of respondents with levels of clinical measurements associated with vascular risk. There was a striking difference in mean body mass index between men (22.4 kg/m^2 ^95% CI 21.5, 23.3) and women (27.2 kg/m^2^, 95% CI 26.1, 27.4), and in abdominal obesity (Table 4), but these differences were not reflected in the way that would be expected in the mean blood pressure, where the systolic pressure was a little higher in the men (136 mmHg, 95%CI 131, 142) than in the women (132 mmHg, 95%CI 131, 134) (Table 4). This relationship was unchanged by adjusting for age. There was no significant difference between men and women in the proportion with high blood pressure (data not shown).

**Table 4:** Mean (95% confidence interval) of clinical measurements by gender.

**Clinical measurement**	**Male**	**Female**
Height (cms) N* = 81 M 277 F	171 (170, 173)	159 (158, 160)
Weight (kgs) N = 83 M 280 F	66.2 (63.5, 68.8)	67.8 (66.0, 69.6)
Body mass index (kg/m^2^) N = 80 M 275 F	22.4 (21.5, 23.3)	26.8 (26.1, 27.4)
Waist circumference N = 79 (M) 278 (F)	82.5 (80.3, 84.8)	83.6 (82.2, 85.0)

Systolic blood pressure (mmHg) N = 86 M 273 F	136 (131, 142)	132 (129, 136)
Diastolic blood pressure (mmHg) N = 86 M 273 F	81 (78, 84)	80 (78, 82)

Ankle Brachial Index (ABI) N = 252 F, 70 M	1.05 (1.01, 1.08)	1.04 (1.03, 1.06),

Total cholesterol (mmol/l) N = 63 M, 203 F	4.28 (4.04, 4.53)	4.53 (4.39, 4.67)
HDL cholesterol (mmol/l) N = 60 M** 203 F	1.47 (1.34, 1.59)	1.44 (1.39, 1.49)

	Percentage (CI)	Percentage (CI)
Proportion with abdominal obesity***	3.80 (0.79, 10.7)	32.7 (27.2, 38.6)
Proportion with ABI <0.9	11.4 (5.07, 21.2)	13.1 (9.19,17.9)

*number of available observations.

** excluding three men with a HDL level at or above 3 mmol/l.

*** Circumference > 102 cms (M), > 88 cms (F) as defined by the National Institute of Health *(Obesity research 1998 6(suppl 2) 51S – 209S)*

**Table 5:** Respondents with clinical measurements associated with vascular risk

**Blood pressure levels and anti-hypertensive treatment**	**Percentage (95% CI)**
Normal blood pressure, no treatment	57.1% (51.8, 62.3)
High blood pressure, no pharmacological treatment	32.6% (27.8, 37.7)
*JNC7 Stage 1 high blood pressure, no pharmacological treatment*	*17.6% (13.8, 21.9)*
*JNC7 Stage 2 high blood pressure, no pharmacological treatment*	*15.0% (11.5, 19.2)*
On pharmacological treatment for high blood pressure	10.3% (7.3, 13.9)
*Normal blood pressure on pharmacological treatment*	*5.29% (3.21, 8.14)*
*JNC7 Stage 1 high blood pressure, on pharmacological treatment*	*2.79% (1.34, 5.06)*
*JNC7 Stage 2 high blood pressure, on pharmacological treatment*	*2.23% (0.967, 4.34)*

**Lipid profile**^**i**^	
Total cholesterol > 5.0 mmol/l	25.6 (20.4, 31.2)
HDL cholesterol < 1.2 mmol/l	22.2 (17.3, 27.7)
HDL:total cholesterol ≥ 20%^ii^	93.5 (89.9, 96.2)

^i ^no-one was using treatment to lower cholesterol levels.

^ii ^excluding 3 men with an HDL cholesterol at or above 3 mmol/l

The mean levels of ABI were similar in men and women, as was the proportion with an ABI at or below 0.9 (Table 4). The proportion with an ABI at or below 0.9 increased sharply with age, reaching 32% in those over 65 years. A fuller report of the ABI findings is available [24].

For comparison with data from other studies, Additional Table 1 (Age and clinical variables) shows the clinical risk factors broken down by 10 year age group.

### Treatment of clinical vascular risk factors

Drugs from the South African essential drug list are available free in local clinics in South Africa, and of the 154 people identified at examination as hypertensive, 37 (24.2%) had used pharmacological treatment for blood pressure in the last week (Table 5). Sixteen were using diuretics, 3 using captopril, reserpine, and atenolol respectively, and 18 using combinations of drugs. Three further people were taking traditional remedies and two were using other products. Of those using pharmacological treatment, 19 (51.4%) had a mean blood pressure reading below 140/90 mm Hg.

No-one was using drugs for treatment of elevated cholesterol levels. South African guidelines [25] set a reading of 5 mmol/l as the highest desirable level of total cholesterol, and just over 25% of participants had levels higher than that (Table 5). Only one person had a cholesterol level above 7.5 mmol/l. However, the HDL/total cholesterol ratio was favourable (> 20%) in over 90% of the population (Table 5).

## Discussion

We have studied risk factors for vascular disease in an older (35 plus) population in rural Southern Africa, and found a pattern that is fairly typical of that hypothesised for populations early in health transition [1,3,26]. High blood pressure is an important clinical issue, affecting 42% of the population. Elevated total cholesterol levels affected about a quarter of the population, but the HDL/total cholesterol ratio was above 20% for over 90% of participants. Our study is the first to report on the distribution of the ABI in a sub-Saharan African population [24]. We found a distribution of ABI that suggests that there is already considerable sub-clinical atheroma in this population, and therefore that the population should be at increased risk of vascular events in future. Heavy cigarette smoking is, as yet, uncommon. We observed an unusual relationship between blood pressure and body size, in that men were markedly slimmer than women but had similar levels of blood pressure.

In South Africa, treatment at community clinics and the drugs prescribed there are free. Nevertheless, the majority of people with high blood pressure (75.8 %) were not taking any treatment. Factors that militate against effective treatment include difficulties with people getting to clinics, difficulties with drug supplies and problems with equipment within clinics [12,27] but there are many other interrelated factors.

### Limitations of this research

The study area was widely dispersed with no tarmac roads. There were long travelling times, delays in repairing equipment and replenishing supplies, and hesitancy from a population with little experience of clinical procedures. The response rate for venepuncture was limited (51%). This was partly due to the hesitancy mentioned above, (which in turn may possibly have been related to a general attitude to allopathic medicine, and therefore a source of bias), but it was also due in large part to the problems we encountered in replenishing supplies in time (and therefore unlikely to be a source of response bias). Nevertheless, our overall response rate was good and the demographic profile of respondents reflected the population reasonably well. The sample size was small, limiting the power of the study.

The study site was in one of the poorer areas of South Africa, but there were more facilities and more state support (disability benefits, pensions) than in most other parts of sub-Saharan Africa. It is likely, therefore, that the levels of risk factors are higher than in the rural parts of other countries in sub-Saharan Africa, but they probably indicate a trend that can be predicted as the economies of other countries develop.

### How do these data compare with other African data?

We found higher rates of hypertension (44% men, 42% women) than those reported in the South African Demographic and Health Survey for (19% men, 21% women) [19]. Our data are comparable in that our nurses were trained by a member of staff from that survey, but the age range of our survey was older (35 years and older compared with 15 years and older in the Demographic and Health Survey) and this would have the effect of increasing the prevalence of hypertension since blood pressure increases with age.

There are few studies available before the 1990's reporting the prevalence of hypertension in non urban black Africans. One study published in 1972 of "South African Rural Bantu" women admitted to one hospital reported that just five of the 485 women studied were hypertensive [28]. However, the authors did not provide any definition of hypertension. Two studies in rural Cameroon and one in a mixed rural and urban population in Nigeria, all published in the late 1990s, reported a prevalence of hypertension (using the same definition of hypertension that we have used) of 15% or less. [29,30] A more recent study (data collected in 2001) in a group of rural and semi- urban villages in Ghana found an overall prevalence of hypertension of 29% in men and women with a mean age of 55 years[6]. However, a study of socially advantaged rural area of Tanzania published in 2000, with data collected in 1997, reported a prevalence of hypertension similar to the current study (41% and 39% in males and females respectively aged 35 to 54 years, and 54% and 61% in those aged 55 years or more[31].

Mean total cholesterol levels in our population were higher than those found from rural areas of North West South Africa, although that population was much younger (15 -65 years, mean 37 years) [32]. Our levels were very similar to those found in the same age range in a socially advantaged part of rural Tanzania [33].

A ratio of HDL cholesterol to total cholesterol of ≥ 20% is protective against heart disease[34], and it has been suggested that the high proportion of black Africans with ratios above this level partly explains the low prevalence of coronary heart disease in South Africa [35]. We found a very high proportion of people with this protective ratio (95%), similar to that found in the Western Cape [36]. We have previously reported little extracranial atherosclerotic disease in stroke survivors in our population[11], and a favourable HDL:TC ratio may be part of the explanation for this.

The levels of obesity were lower than levels reported in urban black people in South Africa [37]. The large difference in obesity between men and women that we observed has been reported, although not explained, throughout South Africa [34,37] but not outside sub-Saharan Africa [38].

### Blood pressure and body size in Africa and the African diaspora

Several studies from sub-Saharan Africa have considered the relationship between body mass index and/or waist circumference and blood pressure. Three studies have explored the slope of the relationship between body mass index and blood pressure in African women [39-41]. All three found non-linear associations, and two of them found that the slope of the relationship between systolic blood pressure and body mass index flattened out in larger women[40,41]. A similar relationship may explain the puzzling differences in body mass index, but not in blood pressure, between men and women in this study.

## Conclusion

Work carried out in the same research site has recently shown that deaths from non-communicable disease have increased by 20% between 1992 and 2003 [42](p44). We have also previously reported a high rate of death from stroke [43]and a high prevalence of survivors of stroke in the research site[11]. These findings, taken together with the findings reported here of a high prevalence of hypertension and of probable sub-clinical atheroma indicate that South Africa is facing the challenge of an emerging epidemic of vascular disease.

This view is supported by other researchers. Murray and colleagues[44] have argued for a 'frameshift in thinking about priorities and public health strategies for less developed regions' (p722), calling for a new emphasis on addressing the burden of vascular disease in low-income as well as high-income countries. They have estimated that, in countries such as South Africa, interventions to address vascular risk will be cost-effective both at the personal level of using drugs to reduce risk, and at the social level of addressing the salt content of food, and providing health education.

Sub-Saharan Africa is already challenged by HIV/AIDS and by the other diseases of poverty. Nevertheless, as the World Health Organisation has argued [45], action to address the emerging threat of non-communicable disease in low and middle income countries is urgently needed. Social level interventions such as salt reduction in food (as a means to reduce population levels of blood pressure) need commitment at national level. Providing individual interventions targeted at vascular disease will require a shift in the paradigm of medical care. Limited resources and skills are a reality, but Sub-Saharan Africa is following the predicted path towards an epidemic of vascular disease, and innovative interventions are needed to avert this outcome.

## Abbreviations

ABI: ankle brachial index

HDL: high density lipoprotein

IPAQ: International Physical Activity Questionnaire

JNC7: seventh Joint National Committee on hypertension

## Competing interests

The author(s) declare that they have no competing interests.

## Authors' contributions

MT, MDC, GLH and ST were all responsible for the study design. MT, GLH and MDC were responsible for the training and supervision of the study nurses. MT and MDC were responsible for supervising data entry and cleaning the data. GF was responsible for the measurement of the ankle brachial index. JM analysed the data. MT wrote the first draft of the paper. All authors read and edited the manuscript and approved the final draft.

## Pre-publication history

The pre-publication history for this paper can be accessed here:



## Supplementary Material

Additional File 1 Mean, standard error and 95% confidence intervals of clinical variables by age group and by gender. shows detailed breakdown of height (cm), weight (kg), body mass index (kg/m^2^), waist circumference (cms), mean systolic blood pressure (mm/Hg), mean diastolic blood pressure (mm/Hg), lowest ankle brachial index, total cholesterol (mmol/l), hdl cholesterol (mmol/l) Click here for file

## References

[B1] Pearson TA (1999). Cardiovascular disease in developing countries: Myths, Realities, and Opportunities. Cardiovascular Drugs and Therapy.

[B2] Cappuccio F (2004). Epidemiologic transition, migration and cardiovascular disease. International Journal of Epidemiology.

[B3] Yusuf S, Reddy S, Stephanie. O, Sonia. A (2001). Global Burden of Cardiovascular Diseases Part I: General Considerations, the Epidemiologic Transisiton, Risk Factors, and the Impact of Urbanization. Circulation.

[B4] Leeder S, Raymond S, Greenberg H (2004). A Race against time.

[B5] World Health Organisation (2002). The World Health Report 2002.

[B6] Cappuccio FP, Micah FB, Emmett L, Kerry SM, Antwi S, Martin-Peprah R, O. PR, Plange-Rhule J, Eastwood JB (2004). Prevalence, detection, management, and control of hypertension in Ashanti, West Africa.. Hypertension.

[B7] Reed DM (1990). The paradox of high risk of stroke in populations with low risk of coronary heart disease.. American Journal of Epidemiology.

[B8] Walker R (1994). Hypertension and stroke in sub-saharan Africa. Transactions of the Royal Society of Tropical Medicine & Hygiene.

[B9] Walker RW, McLarty DG, Kitange HM, Whiting D, Masuki G, Mtasiwa DM, Machibya H, Unwin N, Alberti K, George MM (2000). Stroke mortality in urban and rural Tanzania. The Lancet.

[B10] Kahn K, Tollman SM, Garenne M, Gear JSS (1999). Who dies from what? Determining cause of death in South Africa's rural north-east. Tropical Medicine and International Health.

[B11] SASPI Team (2004). Prevalence of stroke survivors in rural South Africa: results from the Southern Africa Stroke Prevention Initiative (SASPI) Agincourt field site.. Stroke.

[B12] SASPI Team (2004). Secondary prevention of stroke - results from the Southern Africa Stroke Prevention Initiative (SASPI) study,Agincourt Field Site.. Bulletin of the World Health Organisation.

[B13] Connor MD, Walker R, Modi G, Warlow C (2007). The burden of stroke in Black populations in sub-Saharan Africa. Lancet Neurology.

[B14] Tollman SM, Herbst K, Garenne M, Johannesburg, Health Sysems Development Unit (1995). The Agincourt demographic and health study: Phase I..

[B15] Kahn K, Tollman SM, Thorogood M, Connor MD, Garenne M, Collinson M, Lewando Hundt G, Menken J (2005). Health transitions in rural South Africa: New understanding, growing complexity.. Aging in Africa: Current and Future Challenges.

[B16] SASPI Team (2004). The social diagnostics of stroke like symptoms: Healers, doctors and prophets in Agincourt, Limpopo Province, South Africa.. Journal Biosocial Science.

[B17] Kahn K, Collinson MA, Hargreaves JR, Clark SJ, Tollman SM, de Savigny D, Debpuur C, Mwageni E, Nathan R, Razzaque R, Setel PW (2005). Socio-economic status and child mortality in a rural sub-district of South Africa. Measuring Health Equity in Small Areas - Findings from Demographic Surveillance Systems.

[B18] Schellenberg JA, Victoria CG, Mushi A, De Savigny D, Schellenberg D, Mshinda H, Bryce J (2003). Inequities among the very poor: health care for children in rural Tanzania. Lancet.

[B19] Medical Research Council (1998). South Africa Demographic and Health Survey 1998.

[B20] National Heart Lung and Blood Institute (2004). The Seventh Report of the Joint National Committee on Prevention, Detection, Evaluation and Treatment of High Blood Pressure.

[B21] Lee A, Price JF, Smith FB, Wijk M, Fowkes FGR (2004). Improved prediction of fatal myocardial infarction using the ankle brachial index in addition to conventional risk factors: the Edinburgh Artery Study. Circulation.

[B22] International Physical Activity Questionnaire (IPAQ).

[B23] StataCorp Stata Statistical Software: Release 8.0.

[B24] Fowkes FGR, Thorogood M, Connor MD, Lewando-Hundt G, Tzoulaki I, Tollman SM (2006). Distribution of a subclinical marker of cardiovascular risk, the ankle brachial index, in a rural African population. European Journal of Cardiovascular Prevention and Rehabilitation.

[B25] Berger GMB, Marais AD (2000). Diagnosis, management and prevention of the common Dyslipidaemias in South Africa-Clinical Guideline 2000. South Afrcian Medical Association and LASSA working group.

[B26] Gillum RF (1996). The Epidemiology of Cardiovascular Disease in Black Americans. N Engl J Med.

[B27] Thorogood M, Connor MD, Lewando-Hundt G, Tollman S (2007). Understanding and managing hypertension in an African sub-district: A multidisciplinary approach. Scandinavian Journal of Public Health.

[B28] Edginton ME, Hodkinson J, Seftel HC (1972). Disease Patterns in a Sout hAfrican Rural Bantu Population. South African Medical Journal.

[B29] Cooper R, Rotimi C, Ataman S, McGee D, Osotimehin B, Kadiri S, Muna W, Kingue S, Fraser H, Forrester T, Bennett F, Wilks R (1997). The prevalence of hypertension in seven populations of west African origin. Am J Public Health.

[B30] Mbanya JC, Minkoulou EM, Salah JN, Balkau B (1998). The Prevalence of hypertension in rural and urban Cameroon. Internal Journal of Epidemiology.

[B31] Edwards R, Unwin N, Mugasi F, Whiting D, Rashid S, Kissima J, Aspray TJ, Alberti KGMM (2000). Hypertension prevalence and care in an urban and rural area of Tanzania. Journal of Hypertension.

[B32] Oosthuizen W, Voster HH, Kruger A, Venger CS, Druger HS, de Ridder JH (2002). Impact of urbanisation on serum lipid profiles - the THUSA survey. South African Medical Journal.

[B33] Swai ABM, McLarty DG, Kitange HM, Kilima PM, Tatalla S, Keen N, Chuwa LM, Alberti KGMM (1993). Low prevalence of risk factors for coronary heart disease in rural Tanzania. International Journal of Epidemiology.

[B34] Mollentze WF (2004). Epidemiological Aspects in South Africa. South African Journal of Diabetes and Vascular Disease.

[B35] Kruger HS, Venter CS, Vorster HH (2001). Obesity in African women in the North West Province, South Africa is associated with an increased risk of non-communicable diseases: the THUSA study. British Journal of Nutrition.

[B36] Steyn K, Jooste PL, Bourne L, Jean. F, Badenhorst CJ, D.E. B (1991). Risk factors for coronary heart disease in the black population of the Cape Peninsula. The BRISK study.. S Afr Med J.

[B37] Puoane T, Steyn K, Bradshaw D, Laubscher R, Fourie J, Lambert V, Mbananga N (2002). Obesity in South Africa: The South African Demographic and Health Survey. Obesity Research.

[B38] Monteiro C, Moura EC, Conde WL, Popkin BM (2004). Socioeconomic status and obesity in adult populations of developing countries: a review. Bulletin of the WHO.

[B39] Kaufman JS, Asuzu MC, Mufunda J, Forrester T, Wilks R, Luke A, Long AE, Cooper RS (1997). Relationship between blood pressure and body mass index in lean populations. Hypertension.

[B40] Bunker C, Ukoli FA, Matthews KA, Kriska AM, Huston SL, Kuller LH (1995). Weight threshold and blood pressure in a lean black population. Hypertension.

[B41] Kerry S, Emmett L, Micah FB, Martin-Peprah R, Antwi S, Phillips RO, Plange-Rhule J, Eastwood JB, P. CF (2005). Rural and semi-urban difference in salt intake, and its dietray sources, in Ashanti. West Africa.. Ethnicity & Disease.

[B42] Kahn K (2006). Dying to make a fresh start. Mortality and health transition in a new South Africa. Public Health and Clinical Medicine.

[B43] Kahn K, Tollman SM (1999). Stroke in rural South Africa - contributing to the little known about a big problem. South African Medical Journal.

[B44] Murray CJL, Lauer JA, Hutubessy RCW, Niessen L, Tomijima N, Anthony. R, M.M. LC, B. ED (2003). Effectiveness and costs of interventions to lower systolic blood pressure and cholesterol: a global and regional analysis on reduction of cardiovascular-disease risk. The Lancet.

[B45] World Health Organisation (2005). Preventing chronic disease: a vital investment: WHO global report.

