# Supplementary material for: A cross-sectional study of vascular risk factors in a rural South African population: data from the Southern African Stroke Prevention Initiative (SASPI)
Source: BMC Public Health. 2007 Nov 13;7:326. doi: 10.1186/1471-2458-7-326 (PMC2206028; doi:10.1186/1471-2458-7-326)
Supplement: Additional File 1 — Mean, standard error and 95% confidence intervals of clinical variables by age group and by gender. shows detailed breakdown of height (cm), weight (kg), body mass index (kg/m2), waist circumference (cms), mean systolic blood pressure (mm/Hg), mean diastolic blood pressure (mm/Hg), lowest ankle brachial index, total cholesterol (mmol/l), hdl cholesterol (mmol/l) [file 1471-2458-7-326-S1.doc]

Mean, standard error and 95% confidence intervals of clinical variables by age group and by gender

Males _

VARIABLE | Mean Std. Err. [95% Conf. Interval]

-------------+------------------------------------------------

height (cm) |

age 35-44 | 170.4 2.292597 165.7952 175.0048

age 45-54 | 172.0421 1.599695 168.829 175.2552

age 55-64 | 172.2545 2.431362 167.371 177.1381

age 65-74 | 167.7429 2.850719 162.017 173.4687

age75plus | 168.8778 1.691628 165.48 172.2755

-------------+------------------------------------------------

weight (kg) |

age 35-44 | 67.42 3.984896 59.4161 75.4239

age 45-54 | 63.54211 2.484635 58.55157 68.53264

age 55-64 | 60.1 3.232224 53.60789 66.59211

age 65-74 | 66.27143 4.019815 58.19739 74.34546

age75plus | 64.26667 2.16532 59.91749 68.61584

-------------+------------------------------------------------

body mass index (kg/m^2^) |

age 35-44 | 23.17057 1.042987 21.07567 25.26547

age 45-54 | 21.48669 .8237231 19.8322 23.14119

age 55-64 | 20.21341 .9526535 18.29995 22.12687

age 65-74 | 23.55521 1.294279 20.95557 26.15484

age75plus | 22.53554 .6699817 21.18984 23.88124

-------------+------------------------------------------------

waist circumference (cms) |

age 35-44 | 82.06 3.070928 75.89186 88.22814

age 45-54 | 80.67895 2.168415 76.32356 85.03434

age 55-64 | 78.69091 2.475717 73.71829 83.66353

age 65-74 | 84.41429 2.202534 79.99037 88.83821

age75plus | 81.85556 2.397285 77.04047 86.67064

-------------+------------------------------------------------

mean systolic blood pressure (mm/Hg) |

age 35-44 | 120.6 8.485871 103.5556 137.6444

age 45-54 | 128.7105 5.542145 117.5788 139.8423

age 55-64 | 134.0455 8.415207 117.143 150.9479

age 65-74 | 137.2857 9.920432 117.3599 157.2115

age75plus | 133.5 4.622559 124.2153 142.7847

-------------+------------------------------------------------

mean diastolic blood pressure (mm/Hg) |

age 35-44 | 76.5 5.869412 64.71094 88.28906

age 45-54 | 79.92105 3.112243 73.66993 86.17218

age 55-64 | 80.72727 4.808309 71.0695 90.38505

age 65-74 | 80.35714 4.387095 71.5454 89.16888

age75plus | 76.77778 2.431151 71.89467 81.66089

-------------+------------------------------------------------

lowest ankle brachial index |

age 35-44 | 1.070923 .0238631 1.022993 1.118853

age 45-54 | 1.047977 .0280303 .9916763 1.104277

age 55-64 | 1.033994 .0492859 .9350006 1.132988

age 65-74 | 1.054359 .0474949 .9589627 1.149755

age75plus | .9624589 .0545323 .8529275 1.07199

-------------+------------------------------------------------

total cholesterol (mmol/l) |

age 35-44 | 4.06 .5025933 3.050512 5.069488

age 45-54 | 4.131579 .2488632 3.631722 4.631435

age 55-64 | 4.372727 .3355026 3.69885 5.046604

age 65-74 | 4.685714 .2548843 4.173764 5.197664

age75plus | 4.488889 .2936572 3.899061 5.078717

-------------+------------------------------------------------

hdl cholesterol (mmol/l) |

age 35-44 | .98 .3246537 .3279139 1.632086

age 45-54 | 1.552632 .1406685 1.270091 1.835173

age 55-64 | 1.909091 .2484331 1.410098 2.408084

age 65-74 | 1.685714 .1653691 1.353561 2.017868

age75plus | 1.466667 .1384437 1.188594 1.744739

--------------------------------------------------------------

Females

--------------------------------------------------------------

| Mean Std. Err. [95% Conf. Interval]

-------------+------------------------------------------------

height (cm) |

age 35-44 | 161.0362 1.011132 159.04 163.0324

age 45-54 | 159.8283 .8891871 158.0728 161.5837

age 55-64 | 160.812 2.06069 156.7438 164.8802

age 65-74 | 157.45 1.231491 155.0188 159.8812

_subpop_5 | 154.9444 1.332824 152.3132 157.5757

-------------+------------------------------------------------

weight (kg) |

age 35-44 | 68.92069 1.809076 65.34924 72.49214

age 45-54 | 66.28696 2.298888 61.74853 70.82539

age 55-64 | 66.4 2.303772 61.85193 70.94807

age 65-74 | 60.66818 2.936842 54.87031 66.46605

_subpop_5 | 59.54444 3.175305 53.2758 65.81309

-------------+------------------------------------------------

body mass index (kg/m^2^) |

age 35-44 | 26.7108 .7358224 25.25815 28.16345

age 45-54 | 25.91966 .8600429 24.22178 27.61755

age 55-64 | 25.79709 .9348135 23.9516 27.64259

age 65-74 | 24.43647 1.101193 22.26251 26.61043

_subpop_5 | 24.8886 1.39011 22.14427 27.63294

-------------+------------------------------------------------

waist circumference (cms) |

age 35-44 | 81.37414 1.308295 78.79132 83.95696

age 45-54 | 81.8913 1.832152 78.2743 85.50831

age 55-64 | 83.612 1.765902 80.12578 87.09822

age 65-74 | 80.10455 2.616353 74.93938 85.26971

_subpop_5 | 81.07222 3.201254 74.75235 87.39209

-------------+------------------------------------------------

mean systolic blood pressure (mm/Hg) |

age 35-44 | 119.0948 2.47814 114.2025 123.9871

age 45-54 | 129.4348 4.060907 121.4178 137.4518

age 55-64 | 130.28 5.848866 118.7333 141.8267

age 65-74 | 146.8864 5.95582 135.1285 158.6443

_subpop_5 | 136.1111 7.630408 121.0473 151.175

-------------+------------------------------------------------

mean diastolic blood pressure (mm/Hg) |

age 35-44 | 77.22414 1.84712 73.57758 80.8707

age 45-54 | 79.59783 2.079438 75.49263 83.70302

age 55-64 | 78.66 3.038053 72.66232 84.65768

age 65-74 | 83.70455 3.014873 77.75263 89.65646

_subpop_5 | 75.63889 3.302011 69.12011 82.15767

-------------+------------------------------------------------

lowest ankle brachial index |

age 35-44 | 1.101785 .0165286 1.069154 1.134415

age 45-54 | 1.061901 .0254096 1.011738 1.112065

age 55-64 | 1.092029 .031769 1.029312 1.154747

age 65-74 | .9532922 .0395056 .8753007 1.031284

_subpop_5 | .9612791 .0307019 .9006678 1.02189

-------------+------------------------------------------------

total cholesterol (mmol/l) |

age 35-44 | 4.060345 .1210445 3.82138 4.299309

age 45-54 | 4.634783 .1504331 4.3378 4.931765

age 55-64 | 4.708 .141294 4.429059 4.986941

age 65-74 | 5.009091 .2182089 4.578306 5.439876

_subpop_5 | 5.033333 .2067489 4.625173 5.441494

-------------+------------------------------------------------

hdl cholesterol (mmol/l) |

age 35-44 | 1.365517 .0486075 1.269557 1.461477

age 45-54 | 1.53913 .0624367 1.415869 1.662392

age 55-64 | 1.42 .0658281 1.290043 1.549957

age 65-74 | 1.459091 .0646868 1.331387 1.586795

_subpop_5 | 1.522222 .1008317 1.323162 1.721283

--------------------------------------------------------------
